# Effect of Tui-Na versus positional release techniques on pregnancy-related low back pain in the third-trimester: A randomized comparative trial

**DOI:** 10.1097/MD.0000000000040879

**Published:** 2024-12-13

**Authors:** Aliaa M. Elabd, Shahnaz Hasan, Ahmad H. Alghadir, Omar M. Elabd, Ghada Mohamed Shawky, Amir Iqbal, Yara N. Marwan

**Affiliations:** a Basic Science Department, Faculty of Physical Therapy, Benha University, Benha, Egypt; b Department of Physical Therapy and Health Rehabilitation, College of Applied Medical Sciences, Majmaah University, Al-Majmaah, Saudi Arabia; c Rehabilitation Research Chair, Department of Rehabilitation Sciences, College of Applied Medical Sciences, King Saud University, Riyadh, Saudi Arabia; d Department of Orthopedics and its Surgeries, Faculty of Physical Therapy, Delta University for Science and Technology, Gamasa, Egypt; e Department of Physical Therapy, Aqaba University of Technology, Aqaba, Jordan; f Physical Therapy for Woman Health Department, Faculty of Physical Therapy, Benha University, Benha, Egypt.

**Keywords:** exercise therapy, low back pain, manual therapy, pregnancy, traditional Chinese medicine

## Abstract

**Background:**

Researchers are prioritizing the development of an effective treatment approach for third-trimester pregnancy-related low back pain (LBP), a prevalent and costly disorder. Therefore, this study aimed to examine the effects of Tui-Na (TN) versus positional release techniques (PRT) on third trimester pregnancy-related LBP.

**Methods:**

Fifty pregnant women in their third trimester with low back pain were randomly assigned to 1 of 2 groups for 4 weeks of prescribed treatment (TN or PRT). The primary outcome was LBP intensity. Secondary outcomes included the Oswestry disability index for back disability and the pressure pain threshold of lumbar tender points. Two-way multivariate analysis of variance was used for the data analysis.

**Results:**

Multivariate tests indicated statistically significant effects of group (*F* = 10.062, *P* < .001, partial *η*^2^ = 0.302), time (*F* = 473.5, *P* < .001, partial *η*^2^ = 0.953), and group-by-time interactions (*F* = 4.045, *P* < .001, partial *η*^2^ = 0.148). However, the TN group, when compared to the PRT group, revealed a significant decrease in back disability (*P* < .001, partial *η*^2^ = 0.124) and a significant increase in pressure pain threshold at the Rt and Lt points (*P* = .02 and .001, partial *η*^2^ = 0.055, and 0.108, respectively). Within-group comparisons were significant for all measured variables in both the groups (*P* < .001).

**Conclusion:**

Although both TN and PRT are beneficial treatments for third trimester pregnancy-related LBP, TN leads to more beneficial outcomes.

## 1. Introduction

Low back pain (LBP), a prevalent global issue, significantly impacts pregnant women, especially during the third trimester, due to hormonal, postural, and genetic factors.^[1–4]^ Pregnancy-induced LBP can lead to functional disability, depression/anxiety, and significantly impact daily functioning and quality of life, affecting productivity.^[5–8]^ Pregnancy-related low back pain management options include exercise, physical therapy, stability belts, nerve stimulation, medication, acupuncture, massage, yoga, and relaxation techniques, but cost-effectiveness remains uncertain.^[9–11]^

During pregnancy, some women use complementary and alternative medicine such as massage, spinal manipulation, chiropractic, and osteopathy, but there is limited evidence supporting their use for managing pregnancy-related low back pain.^[12,13]^ Recent evidence supports relaxation massages and manual therapy for LBP during pregnancy, but controversy surrounds the most effective type, necessitating further research on different techniques.^[14–16]^

The positional release technique (PRT), also known as strain counterstrain, is a passive manual manipulation method used to alleviate musculoskeletal pain and dysfunction. The treatment method utilizes the body’s positioning to induce therapeutic.^[17]^ Hypothesized physiological impacts include abnormal neuromuscular activity due to muscle spindles and sympathetic nervous system-influenced local circulation or inflammatory responses.^[18]^ PRT is an effective treatment for musculoskeletal conditions like neck pain, hamstring tightness, and ankle sprain, reducing pain, normalizing muscle function, and enhancing quality of life.^[19–22]^ Further, it has been used in patients with LBP to decrease pain, disability, and to enhance lumbar mobility.^[23,24]^

Tui-Na (TN) is a popular traditional Chinese medicine therapy used for joint pain and muscle weakness, often combined with acupuncture, moxibustion, fire cupping, Chinese herbalism, T’ai chi, and qigong.^[25,26]^ Tui-Na (pinch and pull) is a traditional Chinese medicine therapeutic massage technique used to address qi energy imbalances in the body, focusing on manipulation and mobilization, based on acupressure points and body balance.^[27,28]^ Tui-Na uses 3 massage techniques: massage the soft tissue and stimulate acupoints in the meridian system, joint mobilization for spine injuries, and Qi Gong energy exercises like Yang style Taiji Quan for patient guidance.^[26–29]^ TN involves various manipulations applied to affected areas, including rolling, pressing, spine-pinching, vibrating, and oblique pulling.^[30,31]^

Chinese massage has been found to have anti-inflammatory and analgesic properties, potentially aiding in the management of pain in the elderly population without any significant side effects.^[32]^ A meta-analysis of Tuina-focused Chinese medical therapies showed significant effects on pain and functional status in patients with low back pain, particularly when combined with Chinese herbal medicine and acupuncture.^[33,34]^

There is limited evidence examining the effect of positional release versus TN techniques on pregnancy-related LBP in research and clinical practice. In terms of techniques, indications, and contraindications, there are many similarities between both of them. However, treatment is based on different fundamental approaches: Western medicine targets problems of the organ systems, while Chinese massage treats energy balance issues throughout the body.^[25]^

While both therapies can be beneficial for alleviating muscle tension and improving circulation, Chinese massage stimulates the meridian system channels, allowing energy to flow harmoniously, promoting natural body healing.^[30]^ The authors hypothesized that TN may be more beneficial as it offers a holistic approach to healing that is both safe and effective. Its main goal is to get the body’s balance back. TN uses targeted techniques to address parts of the body that are imbalanced and bring about harmony. This makes it especially useful for addressing long-term pain issues like neck or lower back pain.

Based on the preliminary hypothesis, this study examines the effects of TN versus PRT on back pain intensity measured by the visual analogue scale (VAS), back disability measured by the Oswestry disability index (ODI), and pressure pain threshold (PPT) of trigger points in third trimester pregnancy-related LBP patients. The aim is to offer valuable insights for clinicians to create tailored interventions for a prevalent disorder.

## 2. Materials and methods

### 2.1. Study design

This was a based on a single-blinded, 2-arm, parallel-group, randomized comparative study design.

### 2.2. Study setting and participants

Fifty pregnant women in their third trimester participated in the study. Participants with a diagnosis of pregnancy-related LBP were recruited from the gynecology physiotherapy outpatient clinic of Benha University. Patients aged 20 to 40 were screened for eligibility and underwent a standardized physical examination by an assessor blinded to their allocation.^[10]^ The study, which started on January 15, 2024, and ended on March 15, 2024, was completed in 3 months.

**Figure 1.**
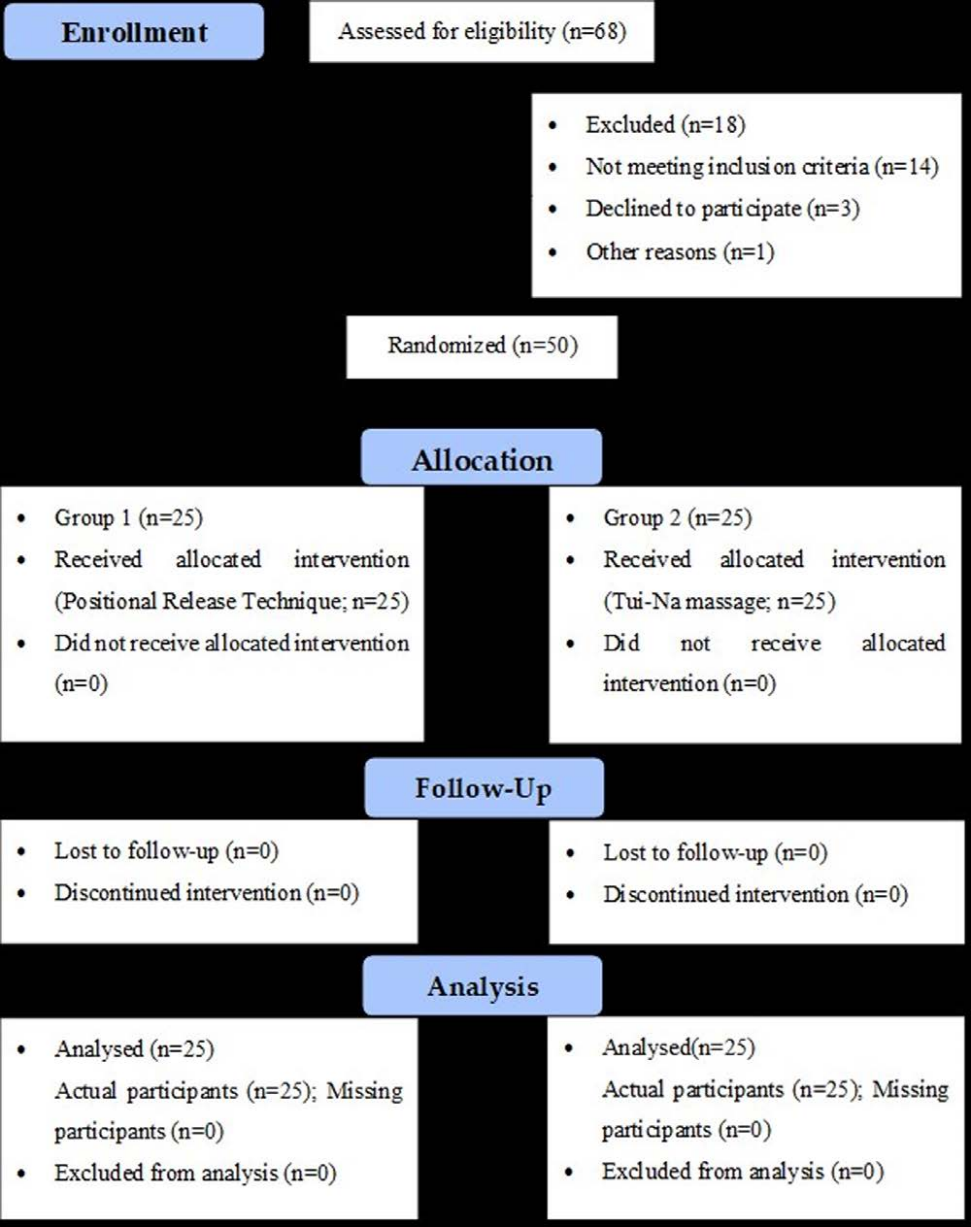
A CONSORT (2010) flow diagram depicts the study procedures, including participants’ enrollment, allocation, follow-up, and analysis.

The study included pregnant women in their third trimester, aged 20 to 40, with a mild to moderate disability score of up to 40% on the ODI.^[35]^ Patients with known spinal pathologies, surgical procedures, neurological disorders, inflammatory disorders, congenital disorders, degenerative alterations, discogenic radiculopathy, current LBP treatment, or visual/auditory problems were excluded. Figure 1, a consolidated standard of reporting trials (2010) flow diagram explains the study procedures.

### 2.3. Randomization/concealed allocation

Patients who met the eligibility criteria were randomly assigned via simple randomization to 1 of 2 groups after baseline assessment: the PRT and TN groups. Hidden allocation was carried out by a researcher who was not involved in patient recruitment or care using a computer-generated random table of numbers created prior to the start of the data collection procedure. Index cards with individual numbers arranged sequentially were folded and placed inside opaque sealed envelopes by the intervention group. The envelope was examined by a second therapist, who was unaware of the findings of the first assessment. In compliance with group assignment, therapy was administered before the initial evaluation day.

### 2.4. Interventions

All patients received 3 sessions of treatment per week for a period of 4 weeks by the same therapist who has more than 10 years of experience. The therapist made his effort to maintain consistency across the sessions. Patients were blinded to their allocation groups.

## 3. PRT group

For the positional release application, the therapist placed the patient in a comfortable position for 90 seconds, for each corresponding muscle, and then passively returned to the starting position.^[36]^ The paraspinal region, the tips of the transverse processes, or the spinous processes are the locations of the posterior lumbar tender points. The patient’s trunk was extended laterally toward the side of the trigger point while lying prone. The patient’s affected leg was supported on her thigh by a therapist who stood on the side of the patient’s pain and placed her knee on the table. Subsequently, the patient’s hip was gently twisted, abducted at a 45-degree angle, and extended. This position was maintained for 90 seconds. Three repetitions were performed with a 90-second break in between. After that, the patient was placed in a comfortable, passive position.^[24]^ Further, when required by the patient, positional release was applied to different muscles such as the quadratus lumborum, iliopsoas piriformis, and gluteus medias.^[37]^

## 4. TN group

**Figure 2.**
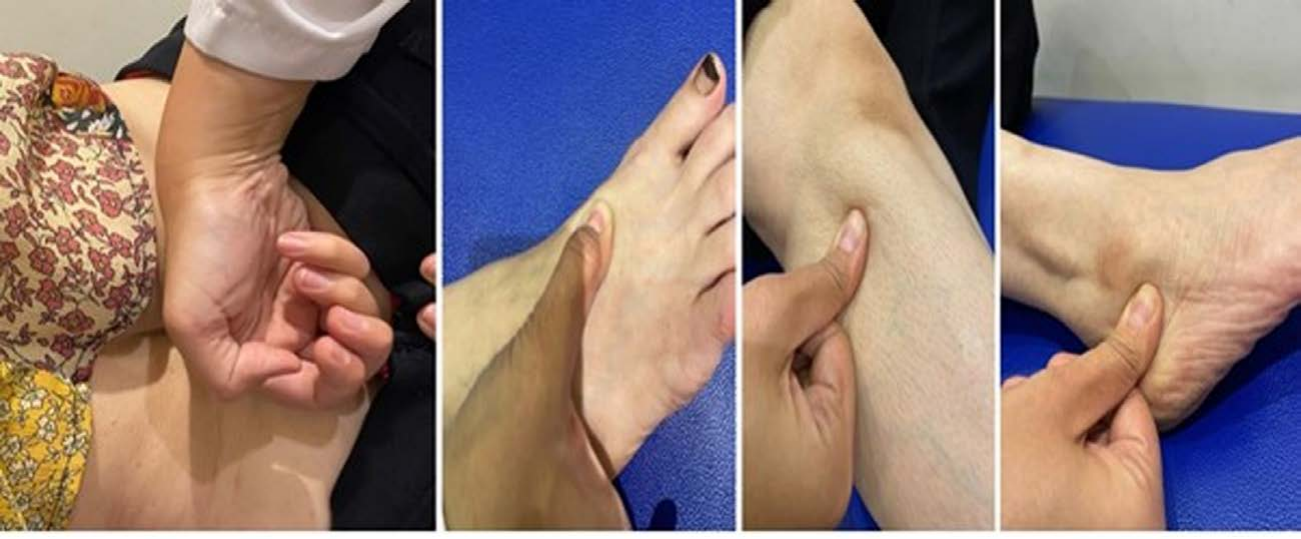
Application of Tui-Na massage.

An experienced therapist applied Tui-Na massage to the lumbar region (proximal points), legs, and feet (distal points). As follow (Fig. 2): First, the therapist initially positioned themselves on the patient’s side, who was positioned side-lying on the treatment table. The procedure involves rolling the bladder meridians on both sides of the spine for 3 to 5 minutes. Second, the therapist used the thumb rock approach on the liver channel point LV3, also known as the Heavenly Star Point, liver channel’s source, and earth point. It is located in a depression distal to the point where the first and second metatarsals join the dorsum of the feet. Third, GB 34 Yang Ling Quan, also known as Yang Mound Spring, which is situated in a depression both anteriorly and inferiorly to the fibula head, was treated with a thumb rock by the therapist. Fourth, the therapist applied a thumb rock to BL62 Shenmai, which is situated in a depression directly below the lateral malleolus, posterior to the peroneal tendon.^[38]^

### 4.1. Outcome measures

The primary outcome measure for this study was back pain intensity, whereas the secondary outcomes were back disability and PPT for lumbar tender points. Outcomes were assessed pre- and posttreatment in both groups, 1 month after the intervention.

VAS was used to evaluate back pain intensity. It is a widely used, reliable, and effective tool.^[39]^ It is a 100-mm horizontal line anchored by “no pain” on the left end and “worst imaginable pain” on the right end as word descriptors. Women marked their pain points on the VAS line, with the score measured from the left end to the marked point.^[40]^

The ODI, consisting of ten items scored on a 0 to 5 scale. The formula (counted marks/50 × 100%) was used for the score calculation. The scores can be interpreted as follows: 0% to 20%, no disability; 21% to 40%, moderate disability; 41% to 60%, severe disability; 61% to 80%, crippled disability; and 81% to 100%, entire disability.^[35,41,42]^

The PPT was measured on both sides of the lumbar spine using a pressure algometer (Wagner, USA). Validity and reliability have been proven.^[43]^ The algometer was inserted into the skin and placed vertically on the tender spots. The most painful point was determined on each side and used for the analysis. Subsequently, a steady rate of 1 kg/cm^2^ of force was applied. The participants were asked to pinpoint the precise moment when they experienced pain. At this point, the threshold displayed on the algometer screen (kg/cm^2^) was recorded. After this technique was repeated 3 times with 10 seconds in between, the mean value was calculated and the participant’s PPT was determined at each of the 2 selected measuring points.^[44,45]^

All outcomes were gathered at baseline and 4 weeks after the intervention by the same knowledgeable assessor who was blinded to the patients’ treatment allocation. The participants were blinded to the findings of their measurements and instructed not to reveal to the assessor whether they had participated in the intervention.

### 4.2. Sample size determination

Calculations for sample size determination were conducted for back pain intensity as a primary outcome measure using G*Power software (version 3.1, Heinrich Heine University, Düsseldorf, Germany). The calculations were based on a 0.487 effect size, an alpha level of.05, and a desired power of 90% from a pilot study involving 10 patients who assigned randomly to 2 equal groups to receive either PRT or TN. The pain intensity values pretreatment in the studied groups were (mean ± standard deviation) 7.5 ± 0.91 and 7.9 ± 1.0, while the posttreatment values were 2.5 ± 0.46 and 3.9 ± 0.7. The *F* test, specifically analysis of variance was chosen to analyze the main effects and interaction effects, with a total sample size of 50 patients to accommodate expected loss.

### 4.3. Ethical considerations

The research ethics review committee at the Faculty of Physical Therapy, Benha University approved this study (approval number: PT.BU.EC.2, dated: 30/11/2023 and registered in clinicaltrial.gov protocol registration and results system (PRS) under identifier number: NCT06198647, dated 10/01/2024 (https://clinicaltrials.gov/study/NCT06198647). This study was conducted at Benha University’s Faculty of Physical Therapy, Benha and followed the Declaration of Helsinki guidelines (2010). All participants returned a signed informed consent form as proof of consent before the start of the study.

### 4.4. Statistical analysis

Version 24 of the IBM statistical package for the social sciences, a computer software program for Windows (IBM Corp. Released 2016. IBM statistical package for the social sciences Statistics for Windows, Version 24.0, Armonk, NY: IBM Corp.) was used for data analysis using intention-to-treat analysis. Five patients with missing post-intervention data were requested to complete an outcome examination shortly after their last session before withdrawal, and the results were incorporated into a statistical analysis. Potential differences in baseline demographic and clinical variables between the groups were examined using an independent sample t test. Two-way multi analysis of variance was used to examine the effects of treatment on outcome measures. For all measurements, the confidence interval was set at 95%.

## 5. Results

Sixty-eight consecutive pregnant females were screened for the eligibility criteria. Fifty patients satisfied the criteria and agreed to participate (age: 32.86 ± 5.29 years, BMI: 23.41 ± 1.62). Patients were randomly divided into 2 groups. PRT group (n = 25; age = 32.80 ± 5.28 years; BMI = 27.77 ± 3.18) and TN group (n = 25; age = 32.92 ± 5.40 years; BMI = 27.9 ± 3.54). No significant differences were found between the groups in either demographic or measured variables at baseline (Table 1).

**Table 1:** Baseline measurements for the studied groups.

Variables	Group A (n = 25) Positional release	Group B (n = 25) Tui-Na	*P* value
Age (years)	32.80 ± 5.28	32.92 ± 5.40	.937 (NS)
Weight (kg)	74.90 ± 9.98	72.86 ± 11.72	.50 (NS)
Height (m)	1.63 ± 0.04	1.62 ± 0.05	.76 (NS)
BMI (kg/m^2^)	28.19 ± 3.19	27.9 ± 3.54	.33 (NS)
Pain (cm)	7.86 ± 0.94	7.96 ± 1.00	.727 (NS)
Disability (%)	40.72 ± 3.79	39.48 ± 3.72	.249 (NS)
PPT for Rt point (kg/cm^2^)	1.92 ± 0.55	2.06 ± 0.65	.401 (NS)
PPT for Lt point (kg/cm^2^)	1.66 ± 0.36	1.76 ± 0.42	.356 (NS)

Data are expressed as mean ± standard deviation.

Lt = left, NS = not significant value if *P* > .05, PPT = pressure pain threshold, Rt = right.

For outcome measures, Table 2 shows the between-group differences with 95% confidence intervals.

**Table 2:** Baseline, post-intervention, and between-group differences and their associated 95% confidence intervals for the outcome measures.

Between groups difference		Posttreatment Mean (SD)	Pretreatment Mean (SD)	Groups
*P*	Mean (95% CI)			
.103 (NS)	0.292 (‐0.06: 0.64)	*Pain*		
		2.84 ± 0.90	7.86 ± 0.94	A
		2.16 ± 0.69	7.96 ± 1.00	B
<.001**	2.54 (1.17: 3.91)	*Disability*		
		26.08 ± 2.96	40.72 ± 3.79	A
		22.24 ± 3.24	39.48 ± 3.72	B
.02*	‐0.218 (‐0.40: ‐0.036)	*PPT for Rt point*		
		2.39 ± 0.24	1.92 ± 0.55	A
		2.68 ± 0.25	2.06 ± 0.65	B
.001*	‐0.308 (‐0.487: ‐0.129)	*PPT for Lt point*		
		2.11 ± 0.50	1.66 ± 0.36	A
		2.62 ± 0.51	1.76 ± 0.42	B

Data are expressed as mean ± standard deviation.

A = control group, B = experimental group, Lt = left, NS = not significant value if *P* > .05, PPT = pressure pain threshold, Rt = right.

* Significant value if *P* < .05.

** Highly significant value if *P* < .01.

Multivariate tests indicated statistically significant effects of group (*F* = 10.062, *P* < .001, partial *η*^2^ = 0.302), time (*F* = 473.5, *P* < .001, partial *η*^2^ = 0.953), and group-by-time interactions (*F* = 4.045, *P* < .001, partial *η*^2^ = 0.148). Univariate tests indicated statistically significant effects of time on all the measured variables (*P* < .001). Regarding group effects, the Tui-Na group, when compared to the positional release group, revealed a significant decrease in back disability (*P* < .001, partial *η*^2^ = 0.124) and a significant increase in PPT at the Rt and Lt points (*P* = .02 and .001, partial *η*^2^ = 0.055, and 0.108, respectively). However, there was no significant change in the pain intensity (*P* = .103, partial *η*^2^ = 0.027). Group-by-time interactions were statistically significant for pain intensity and PPT at the Lt point (*P* = .031 and .026, partial *η*^2^ = 0.047 and 0.050, respectively). However, no significant group-by-time interactions were found for back disability or PPT at the Rt point (*P* = .062 and .423, partial *η*^2^ = 0.036 and 0.007, respectively).

## 6. Discussion

This study was conducted to investigate the effect of PRT versus TN massage on intensity of back pain, back disability, and PPT of lumbar tender points for LBP in third trimester pregnant females. Further, the pre–post effect sizes were large in both intervention groups. In addition to producing results that are both clinically relevant and useful, our treatment option is very affordable and simple to implement, making it a viable option for use in clinical practice.

The results highlight the importance of incorporating various techniques of manual therapy in the management of LBP in the pregnant females. Further, in the current results, both western and eastern concepts used in the studied interventions have been proved beneficial for pregnant females with LBP in their third trimester encouraging their use in clinical practice. However, the results found that the application of TN massage induced an overall significant improvement.

Interestingly, despite the superiority of TN, the results revealed small effect size measurements for group effects on the outcomes which may be attributed to the beneficial effects of PRT as well. Thus, further research is recommended to compare both interventions to nonintervention. Further, the study only examined the short-term effects of the studied interventions. Given the importance of follow-up and long-term studies, especially for pregnancy-related LBP, the authors highly recommend further research studies that examine the long-lasting effect.^[46,47]^

The physiological mechanisms behind the beneficial effects of TN can be attributed to the fact that TN has both physical and energetic effects. Besides its effect for relaxing muscles and tendons, improving local blood circulation, normalizing spinal alignment or balance, and decreasing stress, it also improves qi-blood coordination, regulates Zang-Fu organ functions, warms channels, expels cold, promotes qi circulation, removes blood stasis, and eliminates pathological changes caused by wind cold, qi stagnation, and blood stasis.^[29,30,34]^

The results of this study is aligned with a previous study that suggested the use of TN to loosen muscle adhesions and improve muscle strength in patients with LBP. It can relieve the pathological fatigue of muscles, improve coordination, and restore the physiological balance of muscles and bones.^[48]^ Another study, highlighted the unique curative effect of TN on relaxing skeletal muscles and relieving stress.^[49]^ Therapist-assisted manipulation techniques, including relaxation and adjustment, can reduce nonspecific chronic neck and back pain symptoms, improve anxiety, and enhance patients’ quality of life.

A recent study supported the systematic review by Fan et al., which was conducted to assess the safety and effectiveness of TN for LBP.^[50]^ The study aimed to aid clinicians in practice decisions and advance TN research but faced limitations due to heterogeneity in TN forms and methodological quality. A study’s researchers advised avoiding deep tissue massage during pregnancy to prevent the detachment of blood clots.^[15]^ However, apart from these precautions, relaxation massages can be beneficial during pregnancy to alleviate stress and discomfort such as LBP. In particular, TN massage has been found to reduce depression, anxiety, leg and back pain, lower cortisol levels, and improve immune function.

In a recently published paper, researchers suggested that Tui-Na combined with Yijinjing, a traditional Chinese exercise, significantly lowered the VAS scores and maintained pain relief during a 12-week follow-up period. The Tui-Na combined with Yijinjing group showed significantly reduced disability, anxiety symptoms, tissue hardness, and improved active ranger of motion at the end of the intervention.^[51]^

## 7. Limitations of the study

This study’s main drawback was that the treatments were only used for 4 weeks in order to assess their short-term effects. There was no follow-up to look into the symptoms’ recurrence or long-term effects. Additionally, electromyography was not used to assess the impact on the activity of the lumbar muscles. Furthermore, this study only included patients whose LBP was connected to women in third trimester pregnancy. Therefore, it is not possible to extrapolate the results to other LBP population. Thus, further research is highly recommended to examine the results in other LBP populations or different pregnancy trimesters.

## 8. Conclusion

In third-trimester pregnant females with LBP, both Tui-Na massage and positional release techniques are effective in decreasing back pain intensity, back disability, and tenderness of the lumbar muscles. However, the use of TN resulted in more beneficial overall results. This technique may be a useful treatment option given that it is relatively cost-effective and simple to implement.

## Acknowledgments

The authors extend their appreciation to the Deanship of Scientific Research, King Saud University for funding through the Vice Deanship of Scientific Research Chairs; Rehabilitation Research Chair.

## Author contributions

**Conceptualization:** Aliaa M. Elabd, Shahnaz Hasan, Ahmad H. Alghadir, Omar M. Elabd, Ghada Mohamed Shawky, Amir Iqbal, Yara N. Marwan.

**Data curation:** Aliaa M. Elabd, Yara N. Marwan.

**Formal analysis:** Aliaa M. Elabd, Shahnaz Hasan, Ahmad H. Alghadir, Ghada Mohamed Shawky, Amir Iqbal.

**Investigation:** Shahnaz Hasan, Omar M. Elabd, Yara N. Marwan.

**Methodology:** Aliaa M. Elebd, Omar M. Elabd.

**Supervision:** Ahmad H. Alghadir.

**Writing – original draft:** Aliaa M. Elabd, Shahnaz Hasan, Omar M. Elabd, Ghada Mohamed Shawky, Amir Iqbal, Yara N. Marwan.

**Writing – review & editing:** Aliaa M. Elabd, Shahnaz Hasan, Ahmad H. Alghadir, Omar M. Elabd, Ghada Mohamed Shawky, Amir Iqbal, Yara N. Marwan.
